# An MRI Study of the Metabolic and Structural Abnormalities in Obsessive-Compulsive Disorder

**DOI:** 10.3389/fnhum.2019.00186

**Published:** 2019-06-26

**Authors:** Juliana B. de Salles Andrade, Fernanda Meireles Ferreira, Chao Suo, Murat Yücel, Ilana Frydman, Marina Monteiro, Paula Vigne, Leonardo F. Fontenelle, Fernanda Tovar-Moll

**Affiliations:** ^1^D’Or Institute for Research and Education (IDOR), Rio de Janeiro, Brazil; ^2^Institute of Biomedical Sciences (ICB), Federal University of Rio de Janeiro, Rio de Janeiro, Brazil; ^3^Turner Institute for Brain and Mental Health and School of Psychological Sciences, Monash University, Clayton, VIC, Australia; ^4^Obsessive, Compulsive, and Anxiety Spectrum Research Program, Institute of Psychiatry, Federal University of Rio de Janeiro, Rio de Janeiro, Brazil

**Keywords:** obsessive-compulsive disorder, HMRS, DTI, anterior cingulate cortex, cingulate bundle

## Abstract

Obsessive-compulsive disorder (OCD) is a neuropsychiatric illness characterized by obsessions and/or compulsions. Its pathophysiology is still not well understood but it is known that the cortico-striatal-thalamic-cortical (CSTC) circuitry plays an important role. Here, we used a multi-method MRI approach combining proton magnetic resonance spectroscopy (H1-MRS) and diffusion tensor imaging (DTI) techniques to investigate both the metabolic and the microstructural white matter (WM) changes of the anterior cingulate cortex (ACC) in OCD patients as compared to healthy controls. Twenty-three OCD patients and 21 age-, sex-, and education-matched healthy volunteers participated in the study. Our 1H-MRS findings show increased levels of Glx in ACC in OCD. Further, significantly lower fractional anisotropy (FA) values were observed in OCD patients’ left cingulate bundle (CB) as compared to healthy controls. Finally, there was a negative correlation between FA in the left CB and level of obsessions, as well as the duration of the illness. Our findings reinforce the involvement of CSTC bundles in pathophysiology of OCD, pointing to a specific role of glutamate (glutamine) and WM integrity.

## Introduction

Obsessive-compulsive disorder (OCD) is a neuropsychiatric illness characterized by obsessions and/or compulsions. Obsessions are recurrent, persistent, and unwanted thoughts, urges, or images that generate anxiety and/or distress that are alleviated transiently by compulsions, i.e., repetitive and ritualized behaviors (such as checking, washing, and ordering) or mental acts (such as counting, praying, or repeating words silently; American Psychiatric Association, 2013). Current first-line treatments for OCD include exposure and response prevention (ERP) and serotonin reuptake inhibitors (SRIs; Sookman and Fineberg, 2015). However, as not all patients respond satisfactorily to these treatments, other augmenting drugs (such as glutamate-modulating agents, among others) may need to be added to SRIs (Fineberg et al., 2006; Simpson et al., 2013; Modarresi et al., 2018). Clearly, to develop more effective treatments for OCD, a greater understanding of its etiology and pathophysiology is required.

Although the etiology of OCD remains unclear, research has revealed changes in the cortico-striatal-thalamic-cortical (CSTC) circuits of OCD patients (Pittenger et al., 2011). These circuits link areas that have important roles in the executive function and regulation of behavior (Saxena et al., 2001) and may be well implicated in the mediation of OCD symptoms (Chamberlain et al., 2005). They include cortical and subcortical regions and the white matter (WM) tracts that link them. The cingulate bundle (CB), for example, interconnects the cingulate cortex with limbic regions such as the prefrontal cortex, striatum, and thalamus, and has already been implicated in other neuropsychiatric disorders (Sun et al., 2003; Catheline et al., 2010). The importance of the cingulum in OCD has been highlighted by its use as a target of deep brain stimulation and ablative procedures of treatment refractory OCD patients (Rauch, 2003).

Diffusion tensor imaging (DTI) is a method that allows the measurement of the diffusion characteristics of water molecules *in vivo*. This approach is widely used to investigate WM integrity in psychiatric disorders (Thomason and Thompson, 2011). Although decreased fractional anisotropy (FA) seems disseminated to several brain regions of individuals with OCD, such as the corpus callosum, the longitudinal superior and inferior fasciculus, and the anterior limb of the internal capsule (Szeszko et al., 2005; Bora et al., 2011; Nakamae et al., 2011; Admon et al., 2012), the existing DTI literature suggests the CB to be one of the tracts most consistently affected by decreased WM integrity in adult samples (Piras et al., 2013; Koch et al., 2014). For instance, a recent systematic review found abnormalities in the cingulum in 10 out of the 17 studies (Piras et al., 2013), mostly decreased FA or increased mean diffusivity (MD; consistent with decreased WM integrity; Garibotto et al., 2010; Nakamae et al., 2011). Another proxy for decreased WM integrity was also found in the corticospinal tract, internal capsule, and superior longitudinal fasciculus (Fontenelle et al., 2011).

Glutamate is the principal excitatory neurotransmitter in the brain and a primary neurotransmitter in CSTC circuitry (Shepherd, 2004). It is synthesized from glutamine supplied by astrocytes (Ramadan et al., 2013). Once glutamate is released into the synaptic cleft, it is re-uptaken by astrocytes and converted into glutamine, which will again be used as a precursor of glutamate (Ramadan et al., 2013). Studies using different methods suggest that OCD patients might have a dysfunctional glutamatergic neurotransmission (Carlsson, 2001; Pittenger et al., 2006; Ting and Feng, 2008). For instance, genetic association studies have reported that specific SNPs in or near gene SLC1A1 (which codes for a neural glutamate transporter) such as *rs301443*, rs10491734, and rs7856675 are associated with OCD (Shugart et al., 2009; Samuels et al., 2011). GRIN2B, a gene that codes for a subunit of *N*-methyl-D-aspartate (NMDA) receptors, has also been associated with OCD (Arnold et al., 2009; Kohlrausch et al., 2016). Two studies found elevated cerebrospinal fluid glutamate levels in OCD patients compared to controls (Chakrabarty et al., 2005; Bhattacharyya et al., 2009). There is now evidence of the efficacy of glutamatergic drugs in OCD (Grados et al., 2013; Rodriguez et al., 2013; Marinova et al., 2017). Finally, mice with knocked OUT glutamatergic genes present OCD-like (grooming) behavior (Pittenger et al., 2011).

Perhaps one of the most disseminated methods to assess glutamate and other metabolite levels in the brain is the proton magnetic resonance spectroscopy (H1-MRS). H1-MRS is a noninvasive method that permits *in vivo* quantification of brain biochemistry and has been applied to investigate glutamate levels on OCD. The molecular structures of glutamate and glutamine, which are very similar, give rise to similar magnetic resonance spectra (Ramadan et al., 2013). As a consequence, the combined glutamate and glutamine (Glx) levels are measured by the H1-MRS. The reports, however, have shown some apparent contradictory results. Studies have demonstrated that unmedicated children with OCD had increased Glx levels in the left caudate nucleus that declined after paroxetine treatment as compared to controls (Rosenberg et al., 2000). In adults, a reduction in the anterior cingulate cortex (ACC) Glx levels was restricted to women and negatively correlated with the severity of OCD symptoms (Yücel et al., 2008). Here, we used a multi-method approach combining H1-MRS and DTI techniques to investigate both the metabolic and the microstructural WM changes in OCD patients as compared to healthy controls.

It is important to investigate the relationships between WM integrity and H1-MRS parameters [e.g., glutamate and *N*-acetylaspartate (NAA)] across different neuropsychiatric disorders. For instance, oligodendrocytes (glial cells largely responsible for WM synthesis) seem vulnerable to glutamate receptor-mediated excitotoxicity (McDonald et al., 1998). There is evidence suggesting that changes in NAA may reflect disturbed myelin synthesis (Chakraborty et al., 2001; Madhavarao et al., 2005; Wang et al., 2009; Arun et al., 2010). In healthy adults, WM NAA explained a significant proportion of variability in the FA values, particularly in the splenium of corpus callosum (Wijtenburg et al., 2013). Although a handful of studies have attempted to correlate WM integrity to H1-MRS profile in schizophrenia (Steel et al., 2001; Tang et al., 2007; Rowland et al., 2009; Chiappelli et al., 2015; Reid et al., 2016), the relationship between WM integrity and brain biochemistry in OCD patients remains understudied (Wang et al., 2017, 2018). In the first combined DTI-MRS study, Wang et al. found a positive correlation between FA in the dorsal ACC and choline. In the second, they investigated the anterior thalamic radiation and found a negative correlation between the mean fiber length in the right and ipsilateral thalamic choline level in patients. So far, the association between structural abnormality in the CB and ACC metabolic profile has not been explored. Given the literature reviewed above, we hypothesized: (i) that OCD patients would exhibit decreased FA values in the CB and increased Glx levels in the ACC; (ii) that these findings would correlate with OCD symptomatology; (iii) that they would be independent from medication status; and (iv) that the Glx levels in ACC will negatively correlate with FA in CB.

## Materials and Methods

### Participants

Patients with OCD who were under treatment in the Obsessive, Compulsive, and Anxiety Research Program of the Federal University of Rio de Janeiro and age- and sex-matched healthy community controls participated in the study. All patients met clinical criteria for OCD according to the *Diagnostic and Statistical Manual of Mental Disorders* (*DSM-IV-TR*), had their diagnosis confirmed using the Structured Clinical Interview for the *Diagnostic and Statistical Manual of Mental Disorders* (Del-Ben et al., 2001), and had total Yale–Brown Obsessive–Compulsive Scale (YBOCS; Goodman et al., 1989) scores ≥16. OCD patients and controls with mental retardation, previous suicidal attempts, psychotic disorders, antisocial personality, or contraindications to MRI were excluded from the study. Also, controls with history of obsessions and compulsions were excluded. All participants were older than 18 years and provided their written informed consent to participate in the research protocol, which was approved by the D’Or Institute for Research and Education review board.

### Clinical Assessments

All participants with OCD were interviewed using the YBOCS to evaluate the severity of OCD symptoms. They were also assessed for age at onset (and consequently duration of illness), severity of depression [with the Beck Depression Inventory (BDI; Cunha, 2001)], and functioning [with the *Global Assessment of Functioning* (*GAF*)]. All patients were undergoing pharmacological treatment.

To rate the relative dose of antipsychotic and SRI or other antidepressants being used, scores were attributed to the therapeutically equivalent doses across different medications. According to this scoring system, a score of 1 corresponded to the minimally effective dose for a given SRI, which is also known to occupy at least 80% of the brain serotonin transporters in the striatum (Meyer et al., 2004). Therefore, we feel that the adopted strategy was clinically and biologically valid. Eventually, each participant received an SRI equivalent score, i.e., zero to patients without medication; one to patients who were taking 20 mg of fluoxetine, paroxetine, or citalopram, 50 mg of sertraline, 100 mg of fluvoxamine, or 75 mg of clomipramine; two to patients taking twice the minimally effective doses; and so on and so forth. The score for relative dose of antipsychotic was based on doses equivalent to 100 mg of chlorpromazine (i.e., 1 for patients taking 2 mg of haloperidol, 2 mg of trifluoperazine, 2 mg of pimozide, 2 mg of risperidone, 5 mg of olanzapine, 7.5 mg of aripiprazole, 75 mg of quetiapine, 100 mg of sulpiride, and 1,000 mg of thioridazine, and so on; Woods, 2003).

### Imaging Acquisition

**Figure 1:**
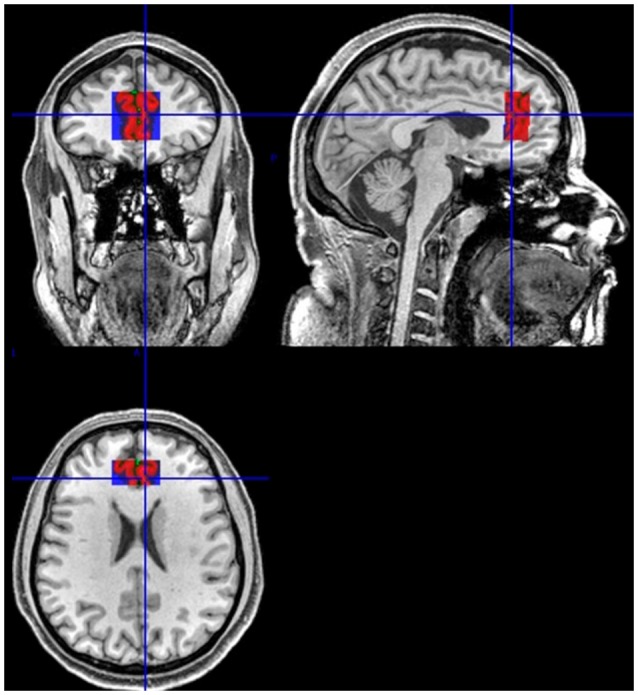
Example of the anatomical reference for the position of the volume of interest (VOI). Sagittal, coronal, and axial views.

Anatomical images were obtained with an Achieva 3T scanner (Philips Medical Systems, Netherlands), using the following pulse sequences: 3D T1-weighted field echo [repetition time (TR)/echo time (TE)/matrix/field of view (FOV) = 7.2 ms/3.4 ms/240 × 240/240 mm, 170 slices, thickness 60 mm] and fluid attenuate inversion recovery [FLAIR; TR/TE/inversion time (TI)/matrix/FOV = 11,000 ms/125 ms/2,800 ms/288 × 168/230 mm, 26 slices, gap = 1 mm, thickness = 4.5 mm]. Diffusion-weighted images (DWIs) were acquired in axial plane with a single-shot, spin-echo echoplanar sequences: TR/3TE/matrix/FOV = 5,582 ms/65 ms/96 × 95/240 × 240 (mm), slice thickness = 2.5 mm, 60 slices without gap. Diffusion sensitization gradients were applied in 32 non-collinear directions, with a *b* factor of 1,000 s/mm^2^. H1-MRS findings were recorded using a point resolved spectroscopy volume selection (PRESS; TE 31 ms/TR 2,000 ms/2,048 points/2 kHz bandwidth). Voxel size was 30 × 30 × 15 mm and placed on the ACC bilaterally (Figure 1). Levels of total N-acetyl-aspartate (NAAt), glutamate and glutamine (Glx), choline (Cho), and creatine + phosphocreatine (Cr) were measured.

### Diffusion Tensor Imaging Procedures

Prior to analysis, participants’ datasets received a numeric code and were divided into controls or OCD patients. All diffusion images were visually inspected for artifacts. Movement artifacts and eddy current distortion effects were corrected. Exclusion criteria included excessive movements and brain lesions. The DTI parameters used to investigate the WM integrity include FA and MD, the frequently used parameters, as they measure the directionality of water diffusion and the magnitude of diffusion, respectively. The diffusion tensor for each voxel was calculated based on the eigenvectors (v1, v2, v3) and eigenvalues (λ1, λ2, λ3). After the FA and MD maps were calculated from the eigenvalues, color-coded maps were generated from the FA values and three vector elements of v1 to visualize the WM tract orientation were performed (DTIFit 2.0, FDT-FMRIB’s *Diffusion Toolbox*, FSL). FA and MD were brain-extracted (BET, DTIFit toolbox, part of FSL 5.0.6, FMRIB software; Smith, 2002) and registered to a common space (Montreal Neurological Institute Template or MNI152) using constrained nonlinear registration (Image Registration Toolkit; Rueckert et al., 1999). The derived FA and MD data were further analyzed using voxelwise whole-brain Tract based Spatial Statistics (TBSS 1.2, FSL; Smith et al., 2006; Simonyan et al., 2008) and Region of interest (ROI) approaches to explore the WM integrity and differences among groups. ROI analyses were selected according to their relationship with OCD pathology and related anatomical changes previously reported.

#### Whole-Brain Analysis

Whole-brain voxelwise statistical analysis of FA and MD were performed using TBSS in order to assess the differences in the WM fiber tracts between OCD patients and healthy volunteers. To preserve the intactness of WM structure, a voxelwise-specific tuned nonlinear registration method was used to register FA and MD images into a standard space (Image Registration Toolkit; Rueckert et al., 1999). Aligned FA images were averaged to create the mean FA from all subjects. The mean FA was used to generate the mean FA “skeleton tract,” which represents the tracts shared by all subjects (Smith et al., 2006). Registered FA data from each subject were “projected” onto the mean FA skeleton mask to generate the final skeletonized FA data.

A threshold was applied (FA >0.2) to restrict the statistical analysis only to WM voxels that were successfully aligned across subjects, maintaining only the subject’s major tract structures. To test for significant local FA and MD differences between controls and OCD, voxelwise cross-subject statistical analysis was carried out using permutation-based non-parametric inference with 10,000 random permutations (FSL Randomize tool) on each voxel of the resulting “mean skeletonized” data (Rueckert et al., 1999) generating the statistical maps The statistical map was “thickened” using spatial smoothing in order to improve visualization.

#### ROI Analysis

ROIs were placed using a DTI–MRI atlas of human WM from Johns Hopkins University (JHU ICBM-DTI-81 White-Matter Labels and JHU WM Tractography Atlas) in the left and right CB. The ROIs were automatically loaded onto the FA and MD maps and visually checked to confirm their location. FA and MD values were automatically extracted using FSL 5.0.6, FMRIB software. Statistical analysis was performed with *p* < 0.05. Analyses including BDI scores and treatment scores as covariates were carried out to investigate associations between the FA and MD values and OCD severity assessed by Y-BOCS scores.

### H1-MRS Procedures

Single-voxel H1-MRS was performed bilaterally and exclusively at the rostral ACC (30 × 30 × 15 mm^3^ fixed). The volume of interest (VOI) was positioned to avoid the skullcap. The anatomical reference for the position of the VOI was the rostrum of the corpus callosum, angulated according to its genu. T2-weighted scans and FLAIR were used to help the placement. Total H1-MRS examination time was approximately 4 min. Eddy current correction was performed for each subject. LCModel (version 6.3- 1H; Provencher, 1993) was used for spectrum quantification. An example of a spectrum is in the Supplementary Material. The amplitude (i.e., the area under the spectra) was firstly fitted for the major metabolites, including NAA, Glx, Cr, and Cho. To minimize changes in magnetic field homogeneity, we used Cr signals as the reference, with the results presented as metabolite-to-Cr ratio, because Cr is relatively stable among other metabolites (Govindaraju et al., 2000). Results are presented in arbitrary units (a.u.). The H1-MRS parameters used for the present study provided robust signals for both the healthy controls and OCD groups in the ACC. The output from LCModel includes the signal-to-noise ratio (SNR) and the mean Cramer–Rao lower bound (CRLB), which is a measure of reliability of the fit. We included participants who had CRLB (SD%) <20% and SNR ≥10. Specifically, healthy controls had an ACC SNR of 21.24 (SD 5.09) and a full width at half maximum peak height (FWHM) of 0.06 ppm (SD 0.02). OCD patients had an SNR of 20.96 (SD 4.89) and an FWHM of 0.05 ppm (SD 0.02). None of these measures were different between the two groups (*p* = 0.86 and 0.26), suggesting that the quality of the data is comparable across the two groups. The CRLB for NAAt, Cr, Cho, and Glx were 5.1%, 3.9%, 4.4%, and 9.5% (SD 2.71, 2.28, 1.89, and 2.73), respectively, for healthy controls, and 4.3%, 3.1%, 3.8%, and 8.3% (SD 2.42, 1.94, 1.69, and 2.29), respectively, for patients.

### Statistics

The *t*-test was used to compare the means of age, schooling, GAF, and BDI among patients and controls. Metabolites were analyzed individually using the SPSS (v.20.0 IBM, Windows). To analyze the associations between H1-MRS metabolite levels and continuous variables (such as the YBOCS, BDI, and GAF scores), Spearman’s correlation coefficients were performed. For FA and MD data processed using the FSL tool, statistical analyses of the voxelwise type of the whole brain were made using non-parametric inference based on permutations, with 10,000 random permutations through the FSL randomization tool, in each voxel contained in the map FA and MD skeletonized mean. Results with a *p*-value of less than 0.05 were considered statistically significant, using family-wise error rate (FWE)-based TFCE (threshold-free cluster enhancement). The *r* values for FA and MD analysis were obtained from *r* = *t*/sqrt(*t*^2^ + df). *t*-values were extracted using randomize (TBSS).

## Results

### Clinical Assessment

A total of 23 OCD patients and 21 healthy volunteers participated in the study. The comparisons between age, sex, years of study, and GAF and BDI scores exhibited by OCD patients and healthy volunteers are shown in Table 1. There was no statistically significant difference between OCD patients and the control group in terms of gender (*p* = 0.239), age (*p* = 0.561), and years of education (*p* = 0.367; Table 1). However, compared to controls, OCD patients scored higher in the BDI (*p* ≤ 0.001) and lower in the GAF (*p* ≤ 0.001). Also, all patients were receiving medication for OCD, including clomipramine (*n* = 10), fluoxetine (*n* = 8), sertraline (*n* = 4); paroxetine (*n* = 3), escitalopram (*n* = 2), and fluvoxamine (*n* = 2). An additional 13 patients were receiving concomitant antipsychotics, seven being typical and six atypical. The mean Y-BOCS scores were 13.78 (3.0) for obsessions, 13.91 (3.1) for compulsions, and 27.70 (5.7) for both symptoms (total score). The mean disease duration was 25.17 (15.9) years.

**Table 1:** Comparison between some sociodemographic and clinical features of obsessive-compulsive disorder (OCD) patients vs. healthy controls.

	OCD (SD)	Controls (SD)	Sig
Age	39.65 (13.7)	37.29 (12.9)	0.561*
Sex (male/female)	15/8	10/11	0.239^†^
Education	14.39 (2.1)	15.00 (2.3)	0.367*
GAF	47.17 (8.1)	92.19 (4.9)	<0.001*
BDI	20.65 (11.0)	4.10 (4.6)	<0.001*
Y-BOCS total	27.70 (5.7)	-	-
Y-BOCS obsessions	13.78 (3.0)	-	-
Y-BOCS compulsions	13.91 (3.1)	-	-
Disease duration	25.17 (15.9)	-	-

GAF, Global Assessment of Functioning; BDI, Beck Depression Inventory; SD, standard deviation; Y-BOCS, Yale–Brown Obsessive–Compulsive Scale. Age, education, and disease duration in years. *t-test; ^†^chi-square test.

### H1-MRS Findings

**Figure 2:**
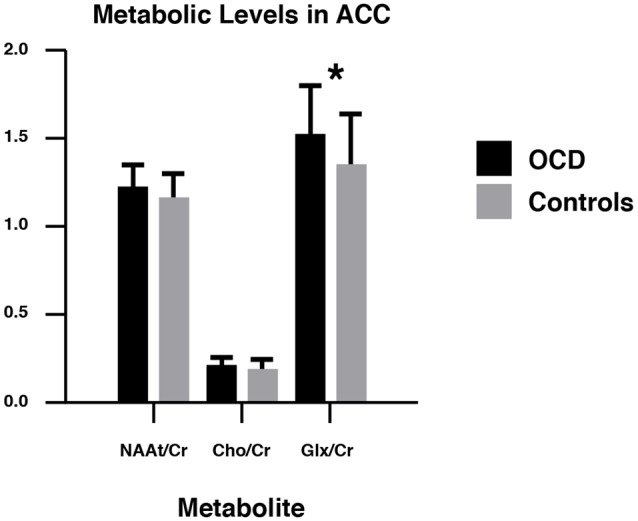
NAAt/Cr, Cho/Cr, and Glx/Cr concentrations in obsessive-compulsive disorder (OCD) patients and healthy controls in the ACC. NAAt, Total *N*-acetyl-aspartate; Cho, choline; Glx, glutamate–glutamine; Cr, creatine + phosphocreatine. ACC, anterior cingulate cortex. *p < 0.05.

In the ACC, NAAt/Cr levels did not significantly differ by group (*p* = 0.191). There were also no significant group differences in Cho/Cr levels (*p* = 0.454). However, compared with controls, OCD patients had significantly higher levels of Glx/Cr in the ACC (*p* = 0.016; Table 2, Figure 2).

**Table 2:** Comparison between metabolites’ concentration in OCD vs. healthy controls in anterior cingulate cortex (ACC).

Metabolite	OCD (*n* = 23)	Controls (*n* = 21)	Sig.
NAAt/Cr	1.18 (0.16)	1.11 (0.16)	0.191
Cho/Cr	0.29 (0.05)	0.28 (0.04)	0.454
Glx/Cr	1.51 (0.27)	1.32 (0.23)	0.016*

NAAt, N-acetyl-aspartate total; Cho, choline; Glx, glutamate–glutamine; Cr, creatine + phosphocreatine. t-test *p < 0.05.

#### Correlation Between H1-MRS Findings and Clinical Data

A correlational analysis was made to investigate if the higher levels of Glx/Cr found in patients were related to symptom severity or disease duration, but no statistically significant results emerged (*p* = 0.931 and *r* = 0.019, and *p* = 0.15 and *r* = 0.31, respectively; see Supplementary Material). As our OCD sample was under pharmacotherapy at the time of the scans, a medication score was created for each group of medication (i.e., SRIs and antipsychotics) according to the equivalent dosage administrated. Then, a correlation analysis was performed between the metabolic ratios and the scores for each patient on each group of medication. Although we were unable to find any significant correlation between different metabolic ratios and the OCD patients’ SRI (*p* = 0.088 and *r* = 0.373 for NAAt/Cr, *p* = 0.119 and *r* = 0.342 for Cho/Cr, and *p* = 0.17 and *r* = 0.303 for Glx/Cr) and antipsychotics’ scores (*p* = 0.073 and *r* = −0.381 for NAAt/Cr, *p* = 0.106 and *r* = 0.346 for Cho/Cr, and *p* = 0.726 and *r* = 0.077 for Glx/Cr), two trends were particularly noticeable, i.e., a positive correlation between the NAAt/Cr and the SRI’s scores (*p* = 0.088 and *r* = 0.373) and a negative correlation between NAAt/Cr levels and antipsychotics’ score (*p* = 0.073 and *r* = 0.381; Supplementary Material). However, these findings did not survive statistical correction. Lack of relationships with medication use was confirmed by comparing the metabolic ratios in the ACC of OCD patients using SRI minus (SRI − ANP) vs. SRI plus antipsychotic (SRI + ANP; see Supplementary Material).

### Diffusion Tensor Imaging Findings

**Figure 3:**
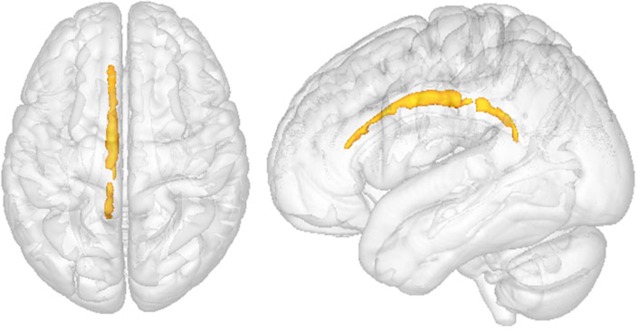
Comparison between OCD patients and healthy controls in the left cingulate bundle (CB). Region of interest (ROI) analysis between groups. Clusters of voxels significantly different (*p* < 0.05) are shown in red for fractional anisotropy (FA) in the white matter (WM). *p* = 0.034.

Voxelwise analysis showed no differences in FA and MD between OCD patients and healthy volunteers. However, ROI analysis showed lower FA in the left CB (*p* = 0.034, Figure 3) of OCD patients compared to healthy controls. Values of MD did not differ significantly between groups.

#### Diffusion Tensor Imaging Findings and Clinical Data

**Figure 4:**
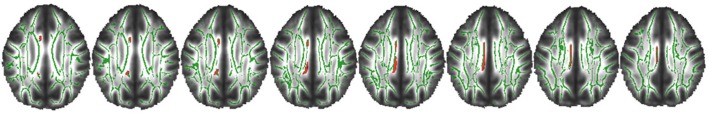
Negative correlation between Y-BOCS obsession score and FA in the left CB. Axial slices. Clusters of voxels significantly different (*p* < 0.05) are shown in red for FA in the WM. *p* = 0.009.

**Figure 5:**
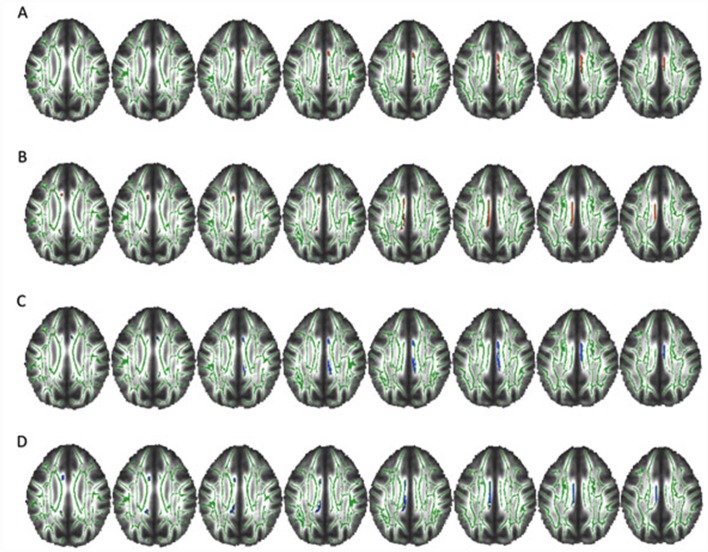
Correlations between FA and mean diffusivity (MD) values and the duration of the illness. Axial slices. Clusters of voxels significantly different (*p* < 0.05) are shown in red for FA and in blue for MD in the WM. **(A,B)** Negative correlation between FA value and years of disease in right (*p* = 0.048) and left (*p* = 0.033) CB. **(C,D)** Positive correlation between MD value and years of disease in right CB (*p* = 0.005) and a trend in left CB (*p* = 0.057).

Significant correlations were found between the severity of symptoms and WM integrity. We found a significant negative correlation between Y-BOCS total score and FA value in left CB (*p* = 0.044 and *r* = 0.510), but it failed to survive the adjustments for depression and treatment score. We also found a significant negative correlation between Y-BOCS obsession subscore and FA value in right CB (*p* = 0.032 and *r* = 0.498) that also failed to survive the covariation analysis. Further, we found a significant negative correlation between Y-BOCS obsession subscore and FA value in left CB (*r* = 0.458; Figure 4). This correlation remained significant after depression (*p* = 0.039), antipsychotics (*p* = 0.010), and SRI scores (*p* = 0.014) were statistically controlled. Regarding the duration of the illness, we found a significant negative correlation with FA values and CB (both left *p* = 0.033 and *r* = 0.494 and right *p* = 0.048 and *r* = 0.551) and positive correlations between MD value and right CB (significant *p* = 0.005 and *r* = 0.498) and left CB (trend *p* = 0.057; Figure 5).

### Correlation Between Structural and Metabolic Data

We investigated the association between Glx/Cr levels in ACC with FA values in the CB but failed to find any significant results (*p* = 0.794 and *r* = −0.041 for left CB; and *p* = 0.560 and *r* = 0.090 for right CB; see Supplementary Information).

## Discussion

We performed a novel and simultaneous investigation of metabolic and structural alterations in OCD patients using 1H-MRS and DTI, respectively. First, metabolic ratios in the ACC in 23 OCD patients and 21 healthy volunteers were compared. The findings revealed higher concentrations of Glx/Cr in OCD patients’ ACC compared to healthy controls (*p* = 0.016). However, Glx/Cr did not correlate with the severity of the symptoms (YBOCS score; *p* = 0.931) or with the duration of the illness (*p* = 0.15). Similarly, two previous studies reported significantly higher Glx levels in unmedicated OCD patients as compared to controls, one in the orbitofrontal cortex (Whiteside et al., 2006) and the other in the ACC (Gnanavel et al., 2014). Thus, 1H-MRS findings in the CB seem to be consistent with the hyperglutamatergic model of OCD, which describes high levels of glutamate in other parts of the CSTC system, such as the orbitofrontal and striatal regions (Rosenberg et al., 2000; Brennan et al., 2013). Indeed, in light of glutamate dysregulation in OCD, there is clinical evidence for the therapeutic utility of glutamate-modulating drugs as an augmentation or monotherapy in OCD patients. These drugs include memantine, anti-convulsant drugs, riluzole, and ketamine (Marinova et al., 2017). Among these, memantine appears to have greater potential (Sheshachala and Narayanaswamy, 2019).

We have also investigated the integrity of the WM in OCD patients compared to healthy controls, firstly by assessing FA and MD through whole-brain TBSS in all WM of OCD patients and controls, and then by placing ROIs on CB (a critical region of the CSTC loop) of the same research subjects. Similarly to previous studies in children and adolescents (Jayarajan et al., 2012; Silk et al., 2013), our whole-brain analysis did not find significant differences between adult patients and controls in FA or MD measurement. However, significantly lower FA values were observed in OCD patients’ left CB as compared to healthy controls in the ROI analysis (*p* = 0.034). Accordingly, reductions of FA in the left anterior cingulate have been reported in both male and female (Lázaro et al., 2014) or just male OCD patients (Ha et al., 2009). Reduced FA values in patients with OCD may indicate changes in myelination or disorganization of fibers within the bundle. Regions that are interconnected by CBs include the prefrontal cortex, the parahippocampal areas, and the striatum (Lochner et al., 2012; Radua et al., 2014). These data suggest microstructural abnormalities in CSTC loops encompassing ACC.

Importantly, we found a negative correlation between FA and severity of obsessions in the left CB (*p* = 0.009), suggesting that the more severe the obsessive symptoms, the lower the integrity of this bundle. Although we could not perform a direct cause-and-effect analysis, this correlation could support the role of the CB (and its related circuits) in the neurobiology of OCD. Reinforcing the involvement of this circuitry in OCD, we also found that the longer the duration of the illness, the lower the FA in CB (*p* = 0.048 for the right CB and *p* = 0.033 for the left CB), suggesting that bundle disorganization is either a consequence of or a risk factor for long-standing OCD. In the same line, disease duration also positively correlated with MD in right CB and, on a trend level, in left CB (*p* = 0.005 and *p* = 0.057, respectively). In addition, the previous report of the lack of WM impairment in younger OCD samples (Jayarajan et al., 2012; Silk et al., 2013) may be consistent with the duration-related impairment of this circuitry in OCD.

Then, we sought to explore whether CB integrity was related to regional neurochemistry. We hypothesized that abnormalities in WM integrity (i.e., reduced FA) would be negatively correlated with higher levels of Glx in patients. More specifically, we predicted that elevated levels of glutamate could lead to excitotoxicity that could influence the integrity of their axons or their connections. However, in contrast to our initial hypothesis, the Glx levels did not correlate with the FA in the CB. A possible reason for the absence of correlation between these parameters is that the increase in Glx may not have been sufficiently large to be neurotoxic.

In order to evaluate the influence of the medication on the metabolic concentrations of the ACC and WM integrity, a dose equivalence score was created for both SRIs and antipsychotics followed by a correlation analysis with the metabolic ratios and DTI values. The analysis showed no significant correlation between the scores and the imaging results. In addition, the sample of patients was separated into two groups: those who used SRIs and those who, besides using SRIs, were also on antipsychotics. Metabolic ratios in the ACC between patient groups were compared, and no meaningful differences were found that could prove the influence of these substances in the metabolite’s ratios. That is, pharmacological treatment with antipsychotics did not seem to affect the H1-MRS variables in our OCD patients, a finding that had already been reported in previous studies in OCD children and adolescents (Ortiz et al., 2015).

Our study has some limitations. First, our OCD patients were under active treatment. Although the inclusion of medicated OCD patients can be considered a major drawback of our study, our analyses took into account the relative dose of medications being used in an attempt to control the effect of SRIs and antipsychotics. Second, possible patients’ comorbidities were not addressed. Therefore, some could ascribe part of our findings to a higher severity of depressive symptoms in OCD patients as compared to healthy controls. However, in contrast to our findings in this OCD cohort, meta-analysis of MRS studies in patients with depression did not show increased but rather decreased glutamate levels in ACC (Luykx et al., 2012). Third, we did not distinguish between glutamate and glutamine levels when using the Glx measure. Yet, the fact that we used a 3.0-T machine, which has greater accuracy in the quantification of metabolites, has probably minimized the effects of this limitation (Paiva et al., 2013).

Finally, the fact that metabolic concentrations were corrected for Cr levels, instead of being absolutely quantified, could also be seen as a systematic limitation. Although the latter methodology is very widely applied and useful for clinical diagnosis (Jansen et al., 2006), metabolic concentrations corrected for Cr levels assume that there are no differences in the levels of Cr between patients and healthy volunteers, a premise that may not necessarily be true, since changes in Cr concentrations have already been reported in other psychiatric disorders such as schizophrenia and bipolar disorder (Ongür et al., 2009).

## Conclusion

Thus, summing up, our findings reinforce the involvement of CSTC and bundles that connect areas within the circuitry in pathophysiology of OCD. Further researches are needed with larger samples taking into account the dimensions of OCD to better understand how these changes correlate with the heterogeneous clinical/phenotypic presentations of OCD.

## Ethics Statement

This study was carried out in accordance with the recommendations of the local ethics guidelines, with written informed consent from all subjects. All subjects gave written informed consent in accordance with the Declaration of Helsinki. The protocol was approved by the ethics committee from D’Or Institute for Research and Education.

## Author Contributions

FT-M, LF, and MY designed the study. IF, PV, JSA, and FF acquired the data, which MM, FF, and CS analyzed. JSA, IF, and FT-M wrote the article, which all authors reviewed and approved for publication.

## Conflict of Interest Statement

The authors declare that the research was conducted in the absence of any commercial or financial relationships that could be construed as a potential conflict of interest.
