# Supplementary material for: An MRI Study of the Metabolic and Structural Abnormalities in Obsessive-Compulsive Disorder
Source: Front Hum Neurosci. 2019 Jun 26;13:186. doi: 10.3389/fnhum.2019.00186 (PMC6620433; doi:10.3389/fnhum.2019.00186)
Supplement: Supplementary file 1 [file Data_Sheet_1.PDF]

frontiers

Supplementary Material

1 Sample MRS spectrum from the rostral ACC region

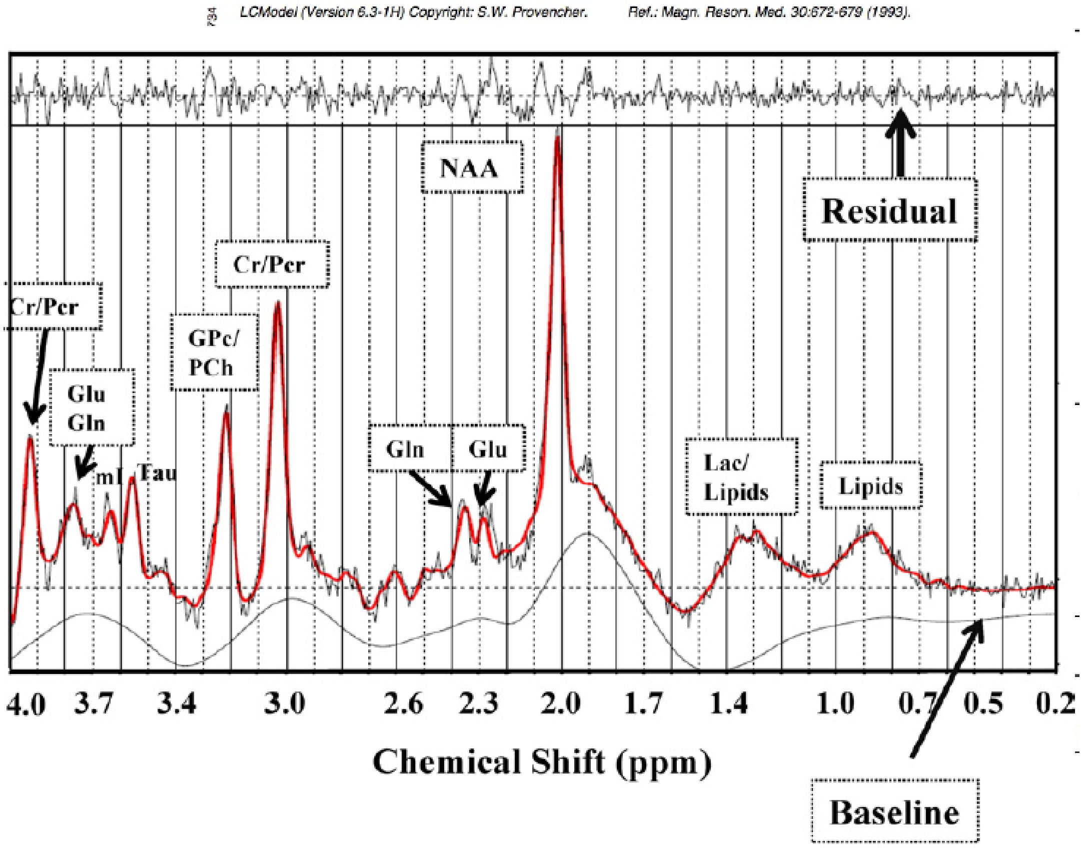

Supplementary Figure 1. An example of a spectrum created by LCModel highlighting main metabolite peaks. NAA: N-acetyl-aspartate; Cho: choline-containing compounds; Glu: glutamate GLN:glutamine; Cr: creatine; Lac: Lactate.

2 Correlation Between Biochemical and Clinical Data

A correlational analysis was made to investigate if metabolite levels were related to symptom severity, disease duration, or medication subscore.

Supplementary table 1. Correlation between metabolites and Y-BOCS scores.

Measure X Metabolites	Pearson R	p Value
**Y-BOCS total**		
NAAt/Cr	-0.032	0.886
Cho/Cr	-0.208	0.341
Glx/Cr	0.019	0.931
**Y-BOCS Obs**		
NAAt/Cr	-0.027	0.903
Cho/Cr	-0.252	0.247
Glx/Cr	-0.066	0.764
**Y-BOCS Com**		
NAAt/Cr	-0.032	0.885
Cho/Cr	-0.138	0.532
Glx/Cr	0.098	0.657

NAAt: N-acetyl-aspartate total; Cho: choline; Glx: glutamate - glutamine; Cr: creatine + phosphocreatine. Y-BOCS (Yale Brown Obsessive-Compulsive Scale). $P<0.05$.

Supplementary table 2. Metabolic ratios in the ACC of OCD patients using SRI vs. SRI plus antipsychotic (SRI+ANP)

Metabolite	SRI + Antipsychotic (n=13)	SRI (n=10)	Sig
NAAt/Cr	1.18 (0.18)	1.18 (0.14)	0.954
Cho/Cr	0.29 (0.04)	0.28 (0.05)	0.735
Glx/Cr	1.56 (0.23)	1.44 (0.30)	0.323

NAAt: N-acetyl-aspartate total; Cho: choline; Glx: glutamate - glutamine; Cr: creatine + phosphocreatine. SRI: serotonin reuptake inhibitors. Mean (SD). T-test

Supplementary table 3. Correlation between illness duration and metabolites

Illness Duration X Metabolites	Pearson R	P Value
NAAt/Cr	-0.323	0.133
Cho/Cr	0.013	0.935
Glx/Cr	-0.31	0.15

NAAt: N-acetyl-aspartate total; Cho: choline; Glx: glutamate - glutamine; Cr: creatine + phosphocreatine. $P<0.05$.

Supplementary table 4. Correlations between metabolic ratios and patients' medication subscores

**SRI's scores**	**Pearson rho**	**P Value**
NAAt/Cr	0.373	0.088
Cho/Cr	0.342	0.119
Glx/Cr	0.303	0.17
**Antipsychotics' score**		
NAAt/Cr	-0.381	0.073
Cho/Cr	0.346	0.106
Glx/Cr	0.077	0.726

NAAt: N-acetyl-aspartate total; Cho: choline; Glx: glutamate - glutamine; Cr: creatine + phosphocreatine. SRI: serotonin reuptake inhibitors. P<0.05

3 Correlation Between Biochemical and structural data

Supplementary table 5. Correlations between Glx/Cr and FA in CB

**FA**	**Pearson rho**	**P Value**
CB Left	- 0.041	0.794
CB Right	0.090	0.560

CB: cingulate bundle. P<0.05
